# Ferredoxin reductase and p53 are necessary for lipid homeostasis and tumor suppression through the ABCA1–SREBP pathway

**DOI:** 10.1038/s41388-021-02100-0

**Published:** 2022-02-04

**Authors:** Yanhong Zhang, Shakur Mohibi, Demitria M. Vasilatis, Mingyi Chen, Jin Zhang, Xinbin Chen

**Affiliations:** 1 Comparative Oncology Laboratory, Schools of Veterinary Medicine and Medicine, University of California at Davis, Davis, CA 95616 USA; 2 Department of Pathology, University of Texas Southwestern Medical Center, Dallas, TX 75390 USA

**Keywords:** Cancer metabolism, Translation

## Abstract

p53 is known to modulate metabolism and FDXR is required for steroidogenesis. Given that FDXR is a target/regulator of p53, the FDXR–p53 axis may play a unique role in lipid metabolism. Here, we found that expression of ABCA1, a cholesterol-efflux pump, was suppressed by loss of FDXR and/or p53, leading to activation of master lipogenic regulators SREBP1/2. Accordingly, lipid droplets, cholesterol, and triglycerides were increased by loss of FDXR or p53, which were further increased by loss of both FDXR and p53. To explore the biological significance of the FDXR–p53 axis, we generated a cohort of mice deficient in *Fdxr* and/or *Trp*53. We found that *Fdxr*^+/−^, *Trp*53^+/−^, and *Fdxr*^+/−^;*Trp53*^+/−^ mice had a short life span and were prone to spontaneous tumors and liver steatosis. Moreover, the levels of serum cholesterol and triglycerides were significantly increased in *Fdxr*^+/−^ and *Trp*53^+/−^ mice, which were further increased in *Fdxr*^+/−^;*Trp53*^+/−^ mice. Interestingly, loss of Fdxr but not p53 led to accumulation of serum low-density lipoprotein. Together, our findings reveal that the FDXR–p53 axis plays a critical role in lipid homeostasis and tumor suppression.

## Introduction

Lipids are essential for life and have many functions, including energy storage, membrane building, and signal transduction [1]. At a normal physiological condition, lipid synthesis is primarily restricted to specialized tissues, such as liver and adipose tissues, while normal cells from other tissues obtain lipids directly from the bloodstream [2]. On the other hand, cancer cells often gain the ability to synthesize lipids and show enhanced lipid uptake [3]. Recent findings showed that reprogramming of lipid metabolism occurs in diabetes mellitus, neurodegeneration, cardiovascular diseases, and cancers [4, 5]. Indeed, aberrant lipid metabolism has been associated with multiple types of cancer [6–11] and increased *de novo* lipid biosynthesis has been recognized as one of the important but not well-characterized hallmarks of cancer cells [12].

Multiple pathways to increase intracellular lipid have been observed in cancers, including increased uptake of extracellular cholesterol through LDL receptor [13–15], decreased expression of ATP-binding cassette A1 protein (ABCA1) [14, 16–19], and aberrant activation of SREBP1/2 [18, 20–22]. ABCA1 is a cholesterol-efflux pump that mediates retrograde sterol movement from the plasma membrane to the ER, and thus modulates maturation of SREBP1/2 [23]. Depletion of cholesterol leads to transportation of SREBPs from the ER to the Golgi apparatus, wherein SREBP1/2 undergo protease cleavage, thereby releasing their active N-terminal domains to the nucleus for transcriptional regulation [24, 25]. SREBP1 regulates genes primarily involved in fatty acid and triglyceride biosynthesis in lipogenic organs, such as the liver, whereas SREBP2 regulates genes predominantly involved in cholesterol synthesis [26].

p53 transcription factor regulates multiple groups of genes associated with metabolism, including oxidative phosphorylation [27, 28], iron and amino acid metabolisms [29, 30], and lipid homeostasis [31, 32]. As a critical modulator of lipid metabolism, p53 is found to regulate ABCA1, malonyl CoA decarboxylase (MCD), LPIN1, G6PD, SIRT1, aromatase, Acad11, DHRS3, and caveolin 1 [28, 32–37]. Thus, the interplay between p53 and lipid metabolism is essential for the decision of cell fate and for tumor suppression.

Biochemically, ferredoxin reductase (FDXR) transports an electron from NADPH to mitochondrial P450 systems via electron shuttle ferredoxin 1 and 2 (FDX1 and FDX2) for biogenesis of Fe–S clusters, bile acids and steroids [38, 39]. It is of note that FDXR and p53 are mutually regulated and that the FDXR–p53 axis plays a critical role in iron homeostasis for tumor suppression [40–42]. However, whether FDXR and the FDXR–p53 axis play a role in lipid metabolism in vivo has not been explored. In this study, we found that like loss of p53, FDXR deficiency led to increased activation of SREBP1/2 and elevated levels of cellular cholesterol and triglycerides via decreased expression of ABCA1. We also found that mice deficient in both *Fdxr* and *Trp53* had a short life span and were prone to spontaneous tumors, liver steatosis and inflammation, as well as elevated levels of serum cholesterol, triglycerides, and low-density lipoprotein.

## Results

### Lack of FDXR, p53, or both leads to altered lipid metabolism via ABCA1–SREBP1/2 pathways

p53 is known to regulate lipid metabolism in part via SREBP2 [18], whereas FDXR is necessary for biosynthesis of bile acid and steroidogenesis [39, 43]. Given that FDXR is a target/regulator of p53 [40–42], the FDXR–p53 axis may play a unique role in lipid metabolism. To test this, we generated a cohort of mouse embryo fibroblasts (MEFs) deficient in *Fdxr*, *Trp*53, or both. We showed that the levels of p53 and p21 proteins were decreased in MEFs deficient in *Fdxr* and/or *Trp53* (Supplementary Fig. S1A). We also showed that the level of p53 transcript remained unchanged, whereas the level of p21 transcript was decreased in *Fdxr*^+/−^, *Trp53*^+/−^, and *Fdxr*^+/−^; *Trp53*^+/−^ MEFs as compared to that in WT MEFs (Supplementary Fig. S1B). These data are consistent with our previous findings that *Fdxr* deficiency leads to decreased expression of p53 and p21 [40]. Additionally, we found that MEFs deficient in *Fdxr* had an increased rate of cell proliferation and that MEFs deficient in both *Fdxr* and *Trp53* had a faster rate of cell proliferation than MEFs deficient in *Fdxr* or *Trp53* alone (Supplementary Fig. S1C), consistent with the observation above that *Fdxr* deficiency led to decreased expression of p53 and p21 (Supplementary Fig. S1A).

**Fig. 1:**
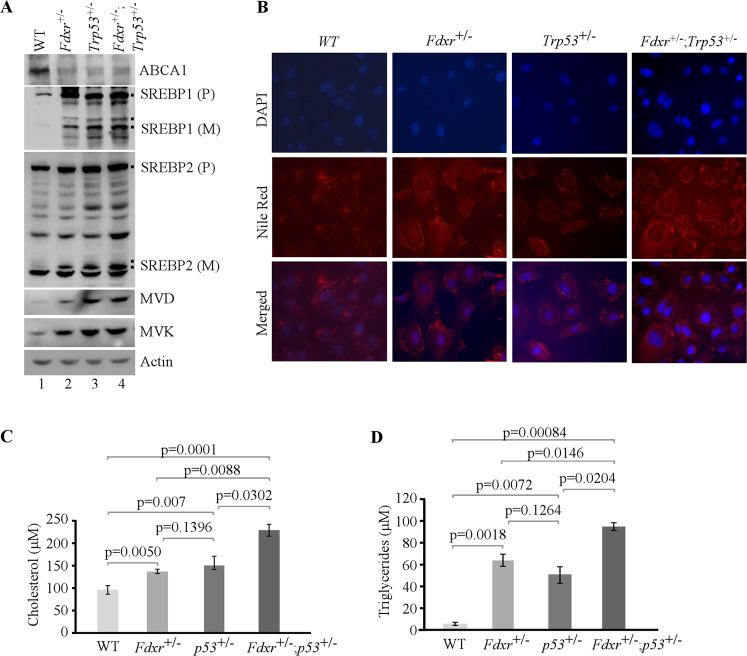
Lack of Fdxr, Trp53, or both leads to altered lipid metabolism though the ABCA1–SREBP pathway in MEFs. **A** The levels of ABCA1, SREBP1/2, MVD, MVK, and actin were measured in WT, *Fdxr*^+/−^, *Trp53*^+/−^, and *Fdxr*^+/−^;*Trp53*^+/−^ MEFs cultured in serum-free media for 4 h. **B** WT, *Fdxr*^+/−^, *Trp53*^+/−^, and *Fdxr*^+/−^;*Trp53*^+/−^ MEFs were cultured in serum-free media for 8 h and then were stained with Nile Red (ex: 488 nm, em: 565 nm). DAPI (ex: 358 nm, em: 461 nm) was used to stain nuclei. **C** Quantitative measurement of intracellular cholesterol. WT, *Fdxr*^+/−^, *Trp53*^+/−^, and *Fdxr*^+/−^;*Trp53*^+/−^ MEFs were cultured in a 96-well plate. After 4 h of fasting, the level of total cholesterol was measured with Cholesterol/Cholesterol Ester-Glo^TM^ assay kit according to the manufacturer’s instruction. Data represent the mean ± SD. **D** Quantitative measurement of intracellular triglyceride. WT, *Fdxr*^+/−^, *Trp53*^+/−^, and *Fdxr*^+/−^;*Trp53*^+/−^ MEFs were cultured in a 96-well plate. After 4 h of fasting, the level of total triglycerides was measured with Triglyceride-Glo^TM^ assay kit according to the manufacturer’s instruction. Data represent the mean ± SD.

To investigate whether loss of FDXR and/or p53 modulates lipid metabolism, MEFs were cultured in a serum-free medium, which mimics depletion of cholesterol response. First, we examined whether Fdxr has an effect on the level of ABCA1, a target of p53 and a cholesterol-efflux pump [18, 23]. We found that Fdxr deficiency alone or together with loss of p53 led to decreased expression of ABCA1 protein (Fig. 1A, compare lane 1 with lanes 2–4), consistent with a previous observation that ABCA1 expression is suppressed by p53 loss [18]. We also found that Fdxr deficiency alone or together with p53 led to increased levels of cleaved nuclear form of SREBP2 (SREBP2-M) (Fig. 1A, compare lanes 2–4 with lane 1), consistent with previous observations that decreased expression of ABCA1 leads to increased cleavage of SREBP2 [18]. Additionally, we measured whether Fdxr has any effect on maturation of SREBP1, which is primarily responsible for synthesis of fatty acid and triglycerides [44, 45]. We found that maturation of SREBP1 was markedly increased by Fdxr deficiency alone or together with p53 (Fig. 1A, compare lane 4 with lanes 2 and 3).

To further explore whether maturation of SREBP2 leads to induction of its targets in the mevalonate pathway, we examined the levels of mevalonate kinase (MVK) and mevalonate decarboxylase (MVD). MVK catalyzes the phosphorylation of mevalonate to produce mevalonate-5-phosphate, whereas MVD catalyzes ATP-dependent decarboxylation of mevalonate pyrophosphate to form isopentenyl pyrophosphate (IPP), both of which are critical enzymes in the mevalonate pathway [46, 47]. We showed that both MVK and MVD were increased by Fdxr deficiency alone or together with p53 (Fig. 1A, compare lane 1 with lanes 2–4), suggesting that the mature form of SREBP2 is active.

To assess whether loss of FDXR, p53, or both alters lipid metabolism, Nile Red (9-diethylamino-5H-benzo[a]phenoxazine-5-one) staining was carried out. We found that lipid droplets were accumulated throughout the cytoplasm in MEFs deficient in *Fdxr*, *Trp53*, or both as compared with WT MEFs (Fig. 1B). Since many intracellular neutral lipids, including triglycerides [48] and sterol esters/cholesterol esters (CE) [49], are recognized by Nile Red, the levels of triglycerides and cholesterol were specifically measured by in vitro enzymatic assays. We found that the intracellular levels of cholesterol and triglycerides were significantly increased by *Fdxr* or *Trp53* deficiency as compared with control (Fig. 1C, D). Additionally, we found that MEFs deficient in both *Fdxr* and *Trp53* had much higher levels of cholesterol and triglycerides than that in MEFs deficient in *Fdxr* or *Trp53* alone (Fig. 1C, D). These data suggest that Fdxr and/or p53 deficiency promotes intracellular lipid accumulation through activation of SREBPs.

**Fig. 2:**
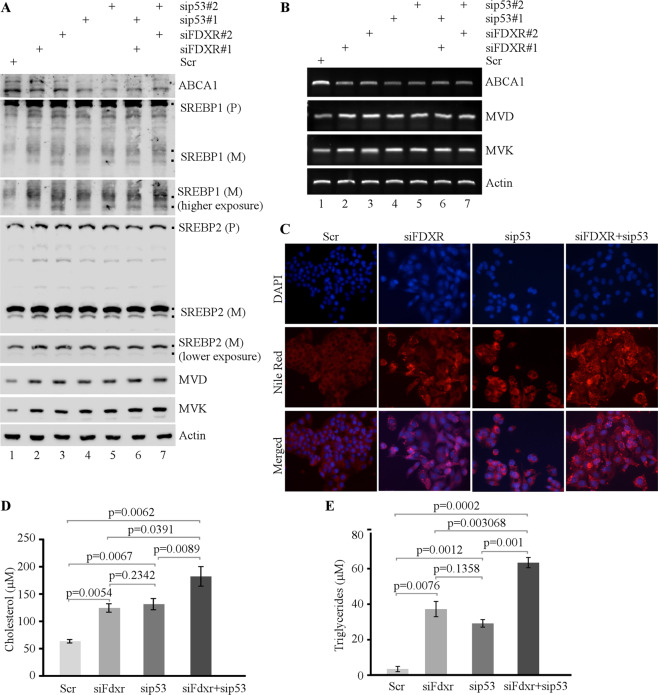
Lack of FDXR, p53, or both leads to deregulation of lipid metabolism through the ABCA1–SREBP pathway in HCC cells. **A** The levels of ABCA1, SREBP1/2, MVD, MVK, and actin proteins were measured in HepG2 cells transfected with scrambled siRNA (Scr) or siRNAs against FDXR and/or p53 for three days, followed by culturing in serum-free media for 4 h. **B** The levels of ABCA1, MVD, MVK, and actin mRNAs were measured in HepG2 cells treated as in A. **C** HepG2 cells transfected with scrambled siRNA (Scr) or siRNAs against FDXR and/or p53 were cultured in serum-free media for 4 h and then stained for lipid droplets with Nile Red (ex: 488 nm, em: 565 nm). DAPI (ex: 358 nm, em: 461 nm) was used to stain nuclei. **D** HepG2 cells transfected with scrambled siRNA (Scr) or siRNAs against FDXR and/or p53 were cultured in a 96-well plate. After 4 h of fasting, the level of total cholesterol was measured with Cholesterol/Cholesterol Ester-Glo^TM^ assay kit according to the manufacturer’s instruction. Data represent the mean ± SD. **E** HepG2 cells transfected with scrambled siRNA (Scr) or siRNAs against *FDXR* and/or *p53* were cultured in a 96-well plate. After 4 h of fasting, the level of total triglycerides was measured with Triglyceride-Glo^TM^ assay kit according to the manufacturer’s instruction. Data represent the mean ± SD.

To determine whether the effect of FDXR and p53 on lipid metabolism observed in murine cells is conserved in human cells, HepG2 and Hep3B hepatocellular carcinoma cell lines were used. HepG2 cells carry wild-type p53, whereas Hep3B is p53-null [50–52]. To test this, FDXR and/or p53 were transiently knocked down in HepG2 cells with siRNAs. We found that the levels of FDXR, p53, and p21 were decreased, whereas cell proliferation was increased upon knockdown of FDXR and/or p53 as compared with control-scrambled siRNA (Supplementary Fig. S2A, B). To confirm this, *FDXR* was knocked out by CRISPR–cas9 in p53-null Hep3B cells and two *FDXR*^+/−^ Hep3B clones (#11 and #15) were generated. Consistently, we found that the level of FDXR was decreased, whereas cell proliferation was increased in *FDXR*^+/−^ Hep3B cells (Supplementary Fig. S3A, B). Next, ABCA1–SREBP1/2 pathways were measured. We found that ABCA1 expression was suppressed, whereas SREBP1/2 maturation and expression of MVD and MVK were increased in HepG2 and Hep3B cells upon loss of FDXR and/or p53 (Fig. 2A and Supplementary Fig. S3C). Consistent with this, ABCA1 mRNA was suppressed, whereas MVD and MVK mRNAs were increased in HepG2 cells upon knockdown of FDXR and/or p53 (Fig. 2B). Moreover, lipid droplets were increased in HepG2 and Hep3B cells upon loss of FDXR and/or p53 (Fig. 2C and Supplementary Fig. S3D). Furthermore, intracellular levels of cholesterol and triglycerides were significantly increased by knockdown of FDXR or p53 alone, which were further increased by combined knockdown of FDXR and p53 in HepG2 cells (Fig. 2D, E). Together, we demonstrated that loss of FDXR and/or p53 leads to activation of SREBP1/2 and increased lipid biosynthesis, which is conserved in murine and human cells.

### Mice deficient in *Fdxr* and *Trp53* were susceptible to spontaneous tumors, liver steatosis, and inflammation

**Fig. 3:**
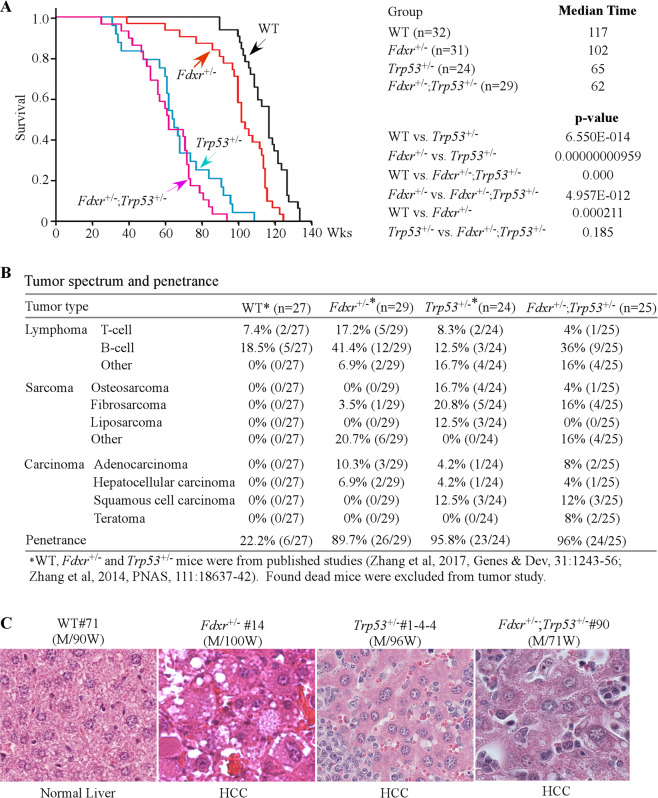
Mice deficient in Fdxr, Trp53, or both have a short life span and are prone to spontaneous tumors. **A** Kaplan–Meier survival curve for *WT*, *Fdxr*^+/−^, *Trp53*^+/−^, and *Fdxr*^+/−^;*Trp53*^+/−^ mice. **B** Tumor spectra and penetrance in *WT*, *Fdxr*^+/−^, *Trp53*^+/−^, and *Fdxr*^+/−^;*Trp53*^+/−^ mice. **C** Representative images of hematoxylin and eosin (H&E)-stained HCC in *Fdxr*^+/−^, *Trp53*^+/−^, and *Fdxr*^+/−^;*Trp53*^+/−^ mice.

Previously, we found that mice deficient in *Fdxr* or *Trp53* had a short life span and were prone to spontaneous tumors [40, 53]. To determine the role of the p53–FDXR axis in tumor suppression, a cohort of WT, *Fdxr*^+/−^, *Trp53*^+/−^, and *Fdxr*^+/−^;*Trp*53^+/−^ mice was generated and monitored for various abnormalities throughout their life. To minimize the number of animals used, the data for WT, *Fdxr*^+/−^, and *Trp53*^+/−^ mice were adapted from previous studies [40, 53, 54]. All the mice were derived from the same C57BL/6 background and maintained in the same facility. We found that the median lifespan for *Fdxr*^+/−^; *Trp*53^+/−^ mice (62 weeks) was significantly shorter than that for WT mice (117 weeks) or *Fdxr*^+/−^ mice (102 weeks), but had no difference from that for *Trp53*^+/−^ mice (65 weeks) (Fig. 3A and Supplementary Tables S1–S4). Histological analysis showed that like *Trp53*^+/−^ (23 out of 24) and *Fdxr*^+/−^ (26 out of 29) mice, *Fdxr*^+/−^; *Trp*53^+/−^ mice (24 out of 25) were tumor-prone as compared with WT mice (6 out of 27) (*p* < 0.0001 by Fisher’s exact test) (Fig. 3B, Supplementary Fig. S4 and Supplementary Tables S1–S4). Interestingly, although both *Fdxr*^+/−^ and *Trp53*^+/−^ mice were prone to spontaneous tumors, their tumor spectra were different. *Fdxr*^+/−^ mice were more prone to lymphomas whereas *Trp53*^+/−^ mice were more susceptible to sarcomas (Fig. 3B and Supplementary Tables S1 and S3). In addition, we found that *Fdxr*^+/−^, *Trp*53^+/−^, and *Fdxr*^+/−^; *Trp*53^+/−^ mice, but not WT mice, developed HCC, albeit at a lower incidence (Fig. 3B, C).

**Fig. 4:**
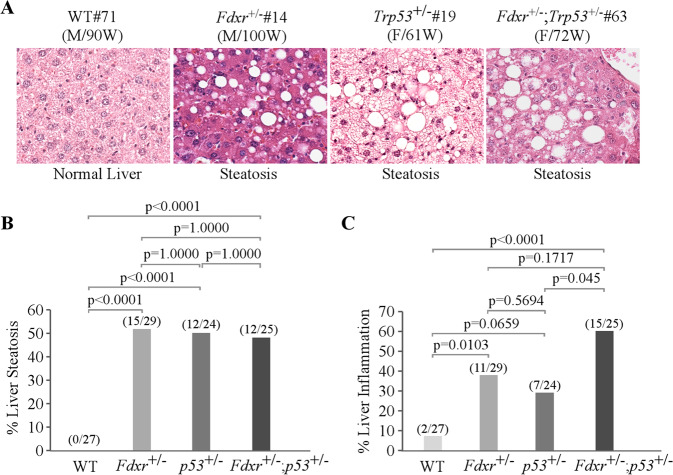
A deficiency in Fdxr, Trp53, or both leads to liver steatosis and chronic inflammation in mouse liver tissues. **A** Representative images of hematoxylin and eosin (H&E)-stained liver in *WT*, *Fdxr*^+/−^, *Trp53*^+/−^, and *Fdxr*^+/−^;*Trp53*^+/−^ mice. **B** The percentage of WT, *Fdxr*^+/−^, *Trp53*^+/−^, and *Fdxr*^+/−^*;Trp53*^+/−^ mice with liver steatosis. **C** The percentage of WT, *Fdxr*^+/−^, *Trp53*^+/−^, and *Fdxr*^+/−^*; Trp53*^+/−^ mice with liver inflammation.

Previously, we showed that some mice deficient in *Fdxr* or *Trp53* were prone to liver steatosis (Fig. 4A, B, Supplementary Tables S1–S3) [40, 53]. Here, we found that *Fdxr*^+/−^; *Trp53*^+/−^ mice were also prone to liver steatosis (Fig. 4A, B, Supplementary Table S4). Since liver steatosis often progresses to steatohepatitis, we examined liver inflammation and showed that *Fdxr*^*+/−*^ and *Fdxr*^+/−^; *Trp53*^+/−^ mice were prone to liver inflammation (Fig. 4C). Additionally, mice deficient in both *Fdxr* and *Trp53* had a higher prevalence of liver inflammation than mice deficient in *Trp53* alone (*p* = 0.045) (Fig. 4C).

### Loss of FDXR and/or p53 leads to activation of SREBP1/2 and high levels of serum cholesterol, triglycerides, and low-density lipoprotein (LDL) in mice

**Fig. 5:**
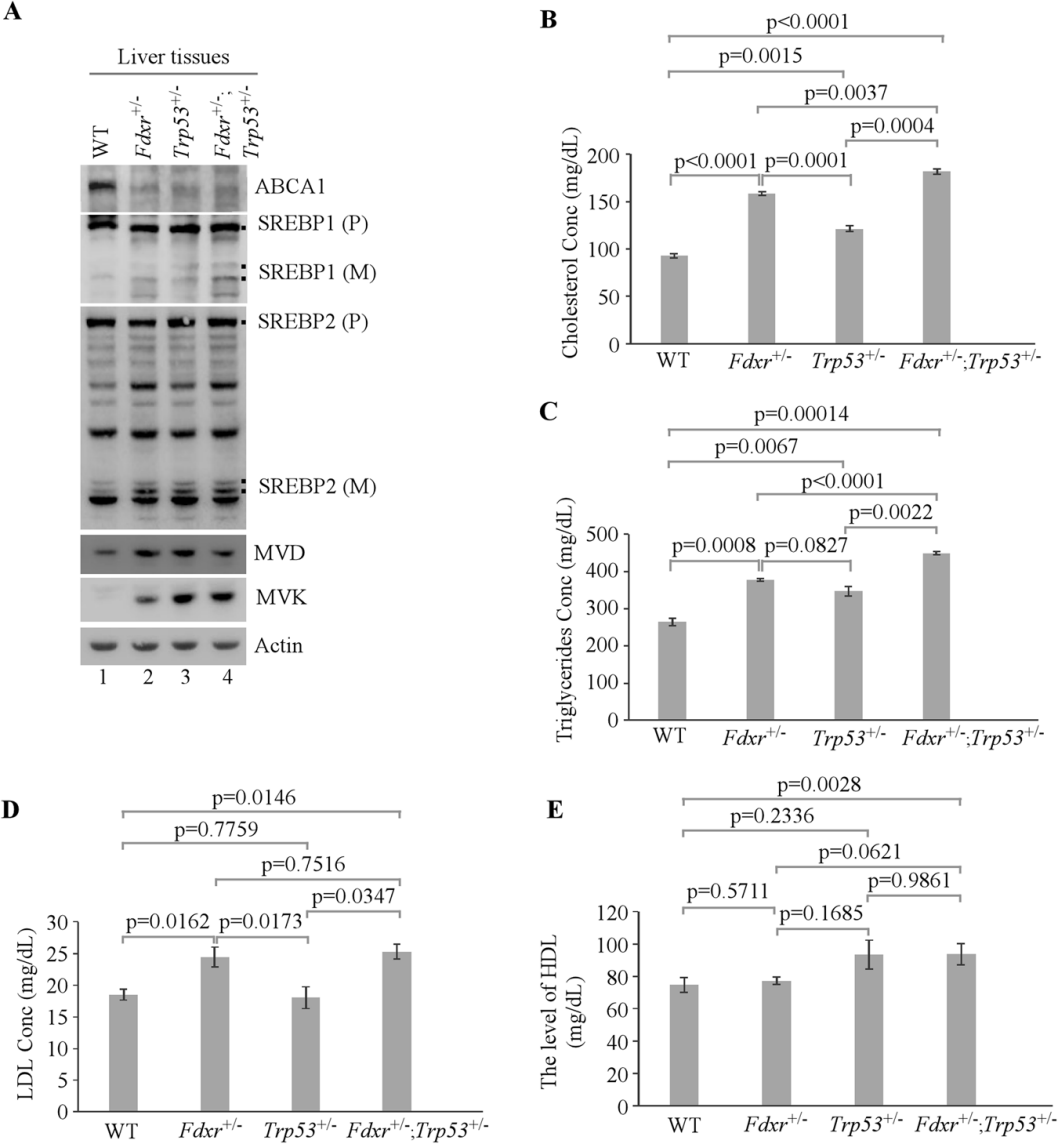
Mice deficient in Fdxr, Trp53, or both are prone to lipid accumulation in the blood. **A** The levels of ABCA1, SREBP1/2, MVD, MVK, and actin were measured in *WT*, *Fdxr*^+/−^, *Trp53*^+/−^, and *Fdxr*^+/−^*;Trp53*^+/−^ mouse liver tissues. **B–E** The levels of blood cholesterol (**B**), triglycerides (**C**), LDL (**D**), and HDL (**E**) in 43-week-old WT, *Fdxr*^+/−^, *Trp53*^+/−^, and *Fdxr*^+/−^;*Trp53*^+/−^ mice (*n* = 5). Values are mean ± SEM and analyzed by 2-tailed *t*-test.

We showed above that following activation of SREBP1/2, intracellular lipid droplets, cholesterol, and triglycerides were increased in MEFs, HepG2, and Hep3B cells (Figs. 1–2 and Supplementary Fig. S3). To determine whether liver steatosis in *Fdxr-* and/or *Trp53-*deficient mice was caused by activation of SREBP1/2, the levels of ABCA1, SREBP1/2, MVD, and MVK were measured in age-matched female mouse livers. Consistently, we found that ABCA1 expression was decreased whereas SREBP1/2 cleavage was increased along with activation of MVD and MVK in *Fdxr*^+/−^, *Trp53*^+/−^, and *Fdxr*^+/−^; *Trp*53^+/−^ liver tissues (Fig. 5A). To further test this, the levels of triglycerides, total cholesterol, low-density lipoprotein (LDL, also called “bad” cholesterol), high-density lipoprotein (HDL, also called “good” cholesterol), and complete blood count (CBC) were measured in blood samples collected from WT, *Fdxr*^+/−^, *Trp*53^+/−^, and *Fdxr*^+/−^; *Trp53*^+/−^ mice (5 for each genotype). We found that loss of Fdxr or p53 did not significantly alter the level of HDL (Fig. 5E) and CBC panels (Supplementary Table S5). However, the levels of blood triglycerides and cholesterol were increased in *Fdxr*^+/−^ and *Trp*53^+/−^ mice (Fig. 5B, C), which were further increased in *Fdxr*^+/−^; *Trp53*^+/−^ mice, suggesting that *Fdxr* deficiency cooperates with *Trp53* deficiency to enhance lipid accumulation. Moreover, we showed that the level of blood LDL was increased in *Fdxr*^+/−^ and *Fdxr*^+/−^; *Trp53*^+/−^ mice but not in *Trp*53^+/−^ mice (Fig. 5D). Since there was no significant change in the level of LDL between *Fdxr*^+/−^ and *Fdxr*^+/−^; *Trp53*^+/−^ mice (Fig. 5D), it suggests that loss of Fdxr is primarily responsible for the accumulation of LDL in both *Fdxr*^+/−^ and *Fdxr*^+/−^; *Trp53*^+/−^ mice.

## Discussion

Biochemically, FDXR is postulated to regulate lipid metabolism through mitochondrial P450 systems for cholesterol side-chain cleavage and hydroxylation of sterol [38, 55–60]. However, it is not clear whether alteration of FDXR activity would actually lead to aberrant lipid metabolism in vivo. In this study, we provided convincing evidence that loss of Fdxr leads to accumulation of cholesterol, triglycerides, and LDL in mouse livers and blood. We also found that loss of FDXR promotes lipid biogenesis by decreasing p53 expression and its target ABCA1, leading to maturation of SREBP1/2 and activation of MVD and MVK. Additionally, excessive accumulation of cholesterol and triglycerides was detected in mice deficient in both *Fdxr* and *Trp53*, suggesting that in addition to downregulation of p53, FDXR may regulate other lipid metabolic pathway(s), which cooperate with p53-dependent pathway to increase lipogenesis. Further supporting this concept is the observation that elevated levels of LDL were detected only in mice deficient in *Fdxr* or both *Fdxr* and *Trp53*, but not in mice deficient in *Trp53* alone (Fig. 5). These results indicate that loss of FDXR enzymatic activity may be responsible for aberrant accumulation of serum lipids and LDL, which merits further investigation.

Accumulating evidence suggests that alterations in lipid metabolism lead to overall metabolic reprogramming in several types of cancer [61], suggesting that it is critical to control lipid metabolism for tumor suppression [18, 62]. Previous reports and our findings here showed that loss of *p53* induces nonalcoholic fatty liver disease (NAFLD) in mice (Figs. 3–5) [63]. Here, we showed that *Fdxr*-deficient mice were prone to liver steatosis and inflammation along with elevated levels of serum lipids, which were aggravated by loss of p53 (Fig. 5). It should be mentioned that (a) germline mutation of *Fdxr* has been reported in multiple families across the globe [64, 65], (b) some polymorphic FDXR variants are unstable [65, 66], and (c) FDXR expression is found to be decreased in a multitude of tumors [40, 67]. As p53 is often mutated in tumors, our study suggests that special attention should be paid to liver function and elevated accumulation of lipids and LDL in cancer patients with FDXR deficiency.

In conclusion, we showed that the FDXR–p53 axis is critical for lipid homeostasis and tumor suppression based on the following evidence [1]: loss of FDXR, p53, or both leads to increased cell proliferation and lipid accumulation [2]; the FDXR–p53 axis regulates maturation of SREBP1/2 via ABCA1 and thus modulates de novo lipid biosynthesis [3]; mice heterozygous in *Fdxr*, *Trp53* or both have a short life span and are prone to spontaneous tumors and liver steatosis [4]; loss of FDXR leads to aberrant accumulation of LDL, serum cholesterol, and triglycerides, the latter two of which were further increased by loss of p53 in mice. Our data indicate that the FDXR–p53 axis could be explored for managing liver disease and a broad spectrum of cancers.

## Materials and methods

### *Fdxr*- and Tr*p53*-mutant mouse models

*Fdxr*^+/−^ mice were generated by the Mouse Biology Program at the University of California at Davis as described previously [40]. *Trp5*3^*+/*−^ mice (on a C57BL/6 background) were purchased from Jackson laboratory. All animals were housed, bred, and maintained in a specific pathogen-free environment at the University of California, Davis. All animal procedures were approved by UC Davis IACUC in adherence to the NIH “Guide for the Care and Use of Laboratory Animals”.

### MEF isolation

*Fdxr* ^+/−^ mice were crossed with *Trp5*3^*+/*−^ mice to generate WT, *Fdxr* ^+/−^, *Trp5*3^*+/*−^, and *Fdxr*^+/−^;*Trp5*3^*+/*−^ MEFs as described previously [40, 68]. The MEFs were cultured in DMEM supplemented with 10% fetal bovine serum (Hyclone Laboratories, Erie, PA), 55 μm β-mercaptoethanol, and MEM nonessential amino acid solution (Cellgro, Manassas, VA).

### Cell culture

HepG2, Hep3B, and their derivatives were cultured in DMEM (Dulbecco’s Modified Eagle’s medium, Invitrogen) supplemented with 10% fetal bovine serum (Hyclone, Logan, UT). HepG2 and Hep3B cell lines were obtained from Dr. Yuyou Duan at UC Davis Medical Center, which were originally purchased from ATCC. Since all cell lines from ATCC have been thoroughly tested and authenticated, we did not retest cells for mycoplasma nor reauthenticate the cell lines. Nevertheless, early passages from original ATCC vials were used within two months. For sterol starvation, cells were rinsed once with serum-free medium and then placed in serum-free medium for 4 h.

### Plasmid construction, cell-line generation, and siRNA transfections

FDXR and p53 guide RNAs (gRNAs) were designed using CRISPR/Cas9 design tool (http://crispr.mit.edu). Cells deficient in *FDXR* or *Tp53* were generated and confirmed as previously described [40]. To generate a vector expressing a single-guide RNA (sgRNA) targeting *FDXR* or *TP53*, two 25-nt oligos were annealed and then cloned into pSpCas9 sgRNA expression vector. The primers used to generate sgRNA expression vectors are listed in Supplementary Table S6. The primers used for genotyping cell lines are listed in Supplementary Table S7.

All small-interfering RNAs (siRNAs) were purchased from Dharmacon RNA Technologies (Chicago, IL). The sequences of the various siRNAs used are listed in Supplementary Table S8. Transfection of siRNA into HepG2 cells was performed using Lipofectamine RNAiMAX transfection reagent (Invitrogen Life Technologies, Grand Island, NY) according to the manufacturer’s protocol.

### Western blot analysis

Western blots were performed as described previously [40]. Antibodies against FDXR, p21, mevalonate decarboxylase (MVD), mevalonate kinase (MVK), and actin were purchased from Santa Cruz Biotechnology (Santa Cruz, CA). Antibody against human/mouse ABCA1 was purchased from Invitrogen Life Technologies (Carlsbad, CA) and Cell signaling Technology (#96292, Danvers, MA). Antibodies against SREBP1 and SREBP2 were purchased from Abcam (Cambridge, MA). HRP-conjugated secondary antibodies against rabbit and mouse IgG were purchased from BioRad (Hercules, CA). The immunoreactive bands were visualized by enhanced chemiluminescence (Thermo Fisher Scientific Inc, Carlsbad, CA) and photographed with the BioSpectrum^®^ 810 Imaging System (UVP LLC, Upland, CA).

### RNA isolation and RT-PCR analysis

Total RNAs were extracted from cells using TRIzol (Invitrogen Life Technologies, Grand Island, NY) according to the manufacturer’s instructions. cDNA was synthesized using RevertAid Reverse Transcriptase Kit (Thermo Fisher Scientific, Grand Island, NY) according to the manufacturer’s protocol. The levels of ABCA1, MVD, MVK, p53, p21, and actin transcripts were measured by RT-PCR. The primers used to amplify ABCA1 were forward primer, 5′-GGA AGA GAC TGC TAA TTG CCA GAC GG -3′, and reverse primer, 5′-GCT GAC AAA TGT GTA CTG TTC GTT GTA CATC -3′. The primers used to amplify MVD were forward primer, 5′-TGA ACT CCG CGT GCT CATC -3′, and reverse primer, 5′-CGG TAC TGC CTG TCA GCT TCT -3′. The primers used to amplify MVK were forward primer, 5′-TGG ACC TCA GCT TAC CCA ACA -3′, and reverse primer, 5′-GAC TGA AGC CTG GCC ACA TC -3′. The primers used to amplify actin (human and mouse), p53 (human and mouse), and p21 (human and mouse) were described previously [40].

### Colony-formation assay

HepG2, Hep3B, or their derivatives (1000 per well) in six-well plates were cultured for 13–15 days. The clones were fixed with methanol/glacial acetic acid (7:1) and then stained with 0.1% of crystal violet.

### Nile Red staining

Nile Red (9-diethylamino-5H-benzo[a]phenoxazine-5-one) was purchased from Cell Signaling Technology (Danvers, MA). Cells were washed with PBS and fixed in 4% paraformaldehyde (Sigma Aldrich, St Louise, MO) for 20 min at room temperature. Nile Red (10 µM in acetone) was diluted into 2 µg/mL and the cells were incubated for 30 min at room temperature. The cells were then counterstained with DAPI and images were collected with fluorescence microscope.

### Measurement of cellular cholesterol and triglycerides

Total cholesterol and triglycerides were determined by an in vitro enzymatic assay. Briefly, cells were plated on a 96-well plate. After 4 h of fasting (cultured in serum-free media), the levels of cholesterol and triglycerides were measured with Cholesterol/Cholesterol Ester-Glow^TM^ assay kit (Promega, Madison, WI) and Triglyceride-Glow^TM^ assay kit (Promega, Madison, WI), respectively.

### Histological analysis

Wild-type, *Fdxr*^+/−^, *Tp53*^+/−^, and *Fdxr*^+/−^;*Tp53*^*+/−*^ mouse tissues were fixed in 10% (w/v) neutral buffered formalin, processed, and embedded in paraffin blocks. Embedded tissues were sectioned (6 µm) and stained with H&E.

### Statistical analysis

The data were presented as mean ± SEM or mean ± SD as indicated. Statistical significance was determined by two-tailed Student’s *t* test. Values of *P* < 0.05 were considered significant. For Kaplan–Meyer survival analysis, log-rank test was performed. Fisher’s exact test was used for comparison of tumors, liver steatosis, and liver inflammation from different genotypes.

## Supplementary information

Supplementary Figures S1-S4 with legends

Supplementary Tables S1-S8
