# Supplementary material for: Ferredoxin reductase and p53 are necessary for lipid homeostasis and tumor suppression through the ABCA1–SREBP pathway
Source: Oncogene. 2022 Feb 4;41(12):1718–26. doi: 10.1038/s41388-021-02100-0 (PMC8933276; doi:10.1038/s41388-021-02100-0)
Supplement: Supplementary file 1 — Supplementary Figures S1-S4 with legends [file 41388_2021_2100_MOESM1_ESM.pdf]

Supplementary Figure S1

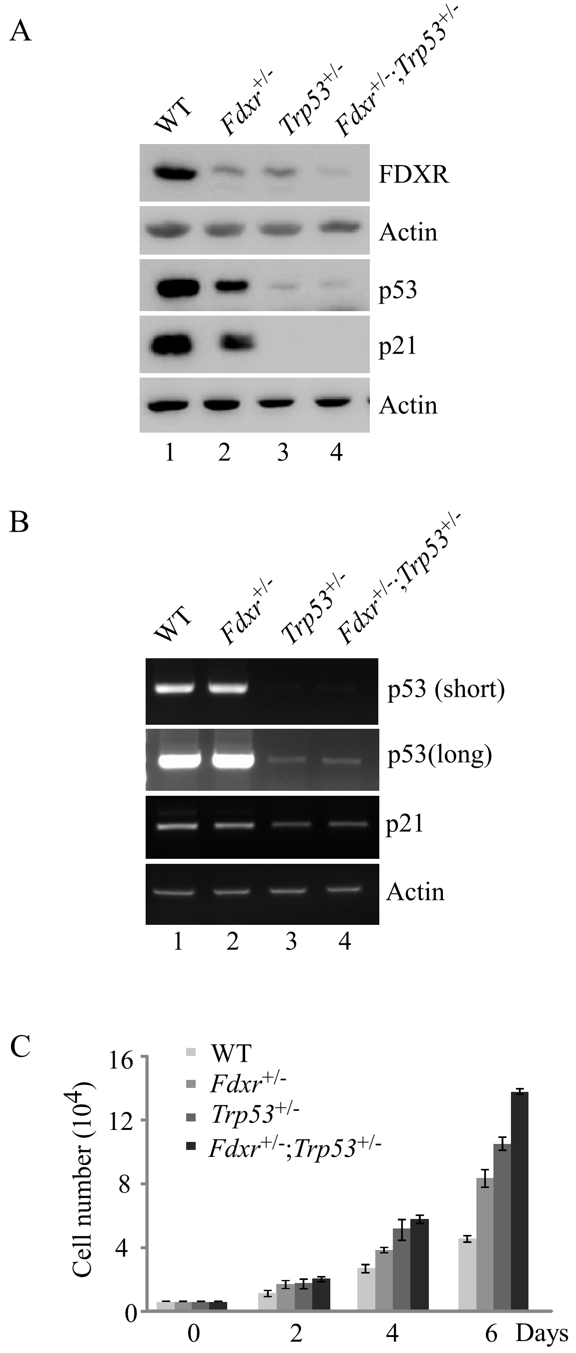

Supplementary Figure S1. Lack of *Fdxr*, *Trp53* or both promotes cell growth

(A) The levels of FDXR, p53, p21, and Actin were measured by western blotting in WT, *Fdxr*$^{+/-}$, *Trp53*$^{+/-}$ or *Fdxr*$^{+/-}$;*Trp53*$^{+/-}$ MEFs.

(B) The levels of p53, p21 and actin transcripts were measured by RT-PCR in WT, *Fdxr*$^{+/-}$, *Trp53*$^{+/-}$ or *Fdxr*$^{+/-}$;*Trp53*$^{+/-}$ MEFs.

(C) The number of WT, *Fdxr*$^{+/-}$, *Trp53*$^{+/-}$, and *Fdxr*$^{+/-}$;*Trp53*$^{+/-}$ MEFs was measured over a 6-day period. The number was presented as mean ± SD from three separate experiments.

Supplementary Figure S2

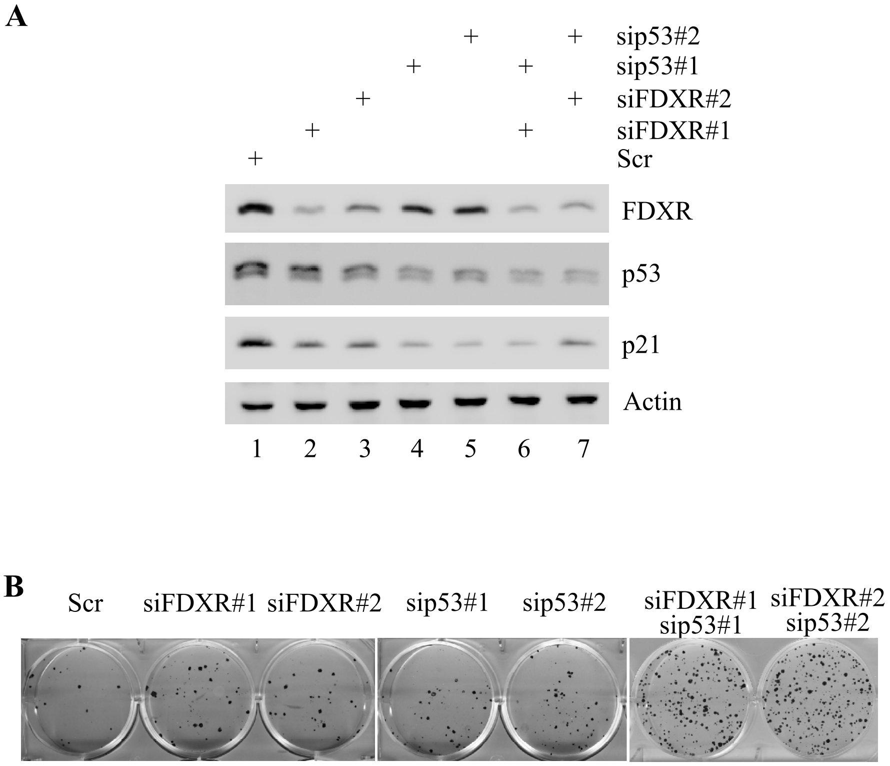

Supplementary Figure S2. A deficiency in *FDXR*, *p53* or both promotes cell growth in HepG2 cells

(A) HepG2 cells were transfected with scrambled siRNA (Scr) or siRNAs against *FDXR* and/or *p53* for 72 h. Cell lysates were collected and subjected to western blot analysis with antibodies against FDXR, p53, p21 and Actin.

(B) Colony formation assay was performed with HepG2 cells transfected with scrambled siRNA (Scr) or siRNAs against FDXR and/or p53.

Supplementary Figure S3

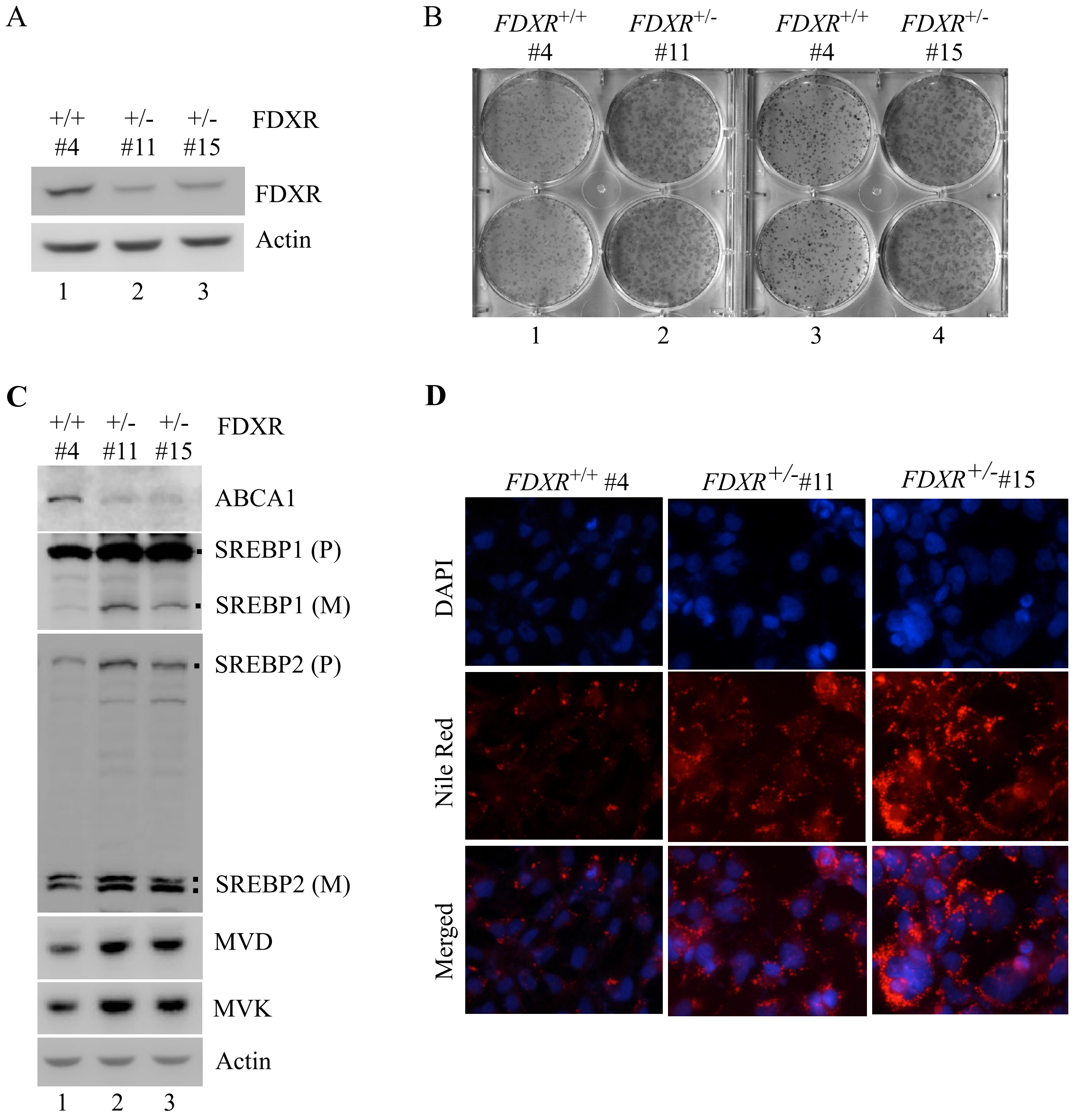

Supplementary Figure S3. Deficiency in *FDXR* promotes cell growth in p53-null Hep3B cells.

(A) The levels of FDXR and Actin were measured by western blotting in isogenic control and *FDXR*$^{+/-}$ Hep3B cells (clone#11 and #15).

(B) Colony formation assay was performed with isogenic control or *FDXR*$^{+/-}$ Hep3B (clone#11 and #15) cells.

(C) The levels of ABCA1, SREBP1/2, MVD, MVK, and Actin were measured in isogenic control and *FDXR*$^{+/-}$ Hep3B (clone#11 and #15) cells cultured in serum-free media for 4 h.

(D) Isogenic control and *FDXR*$^{+/-}$ Hep3B (clone#11 and #15) cells were cultured in serum-free media for 4 h and then followed by Nile Red staining. DAPI was used to stain nuclei.

Supplementary Figure S4

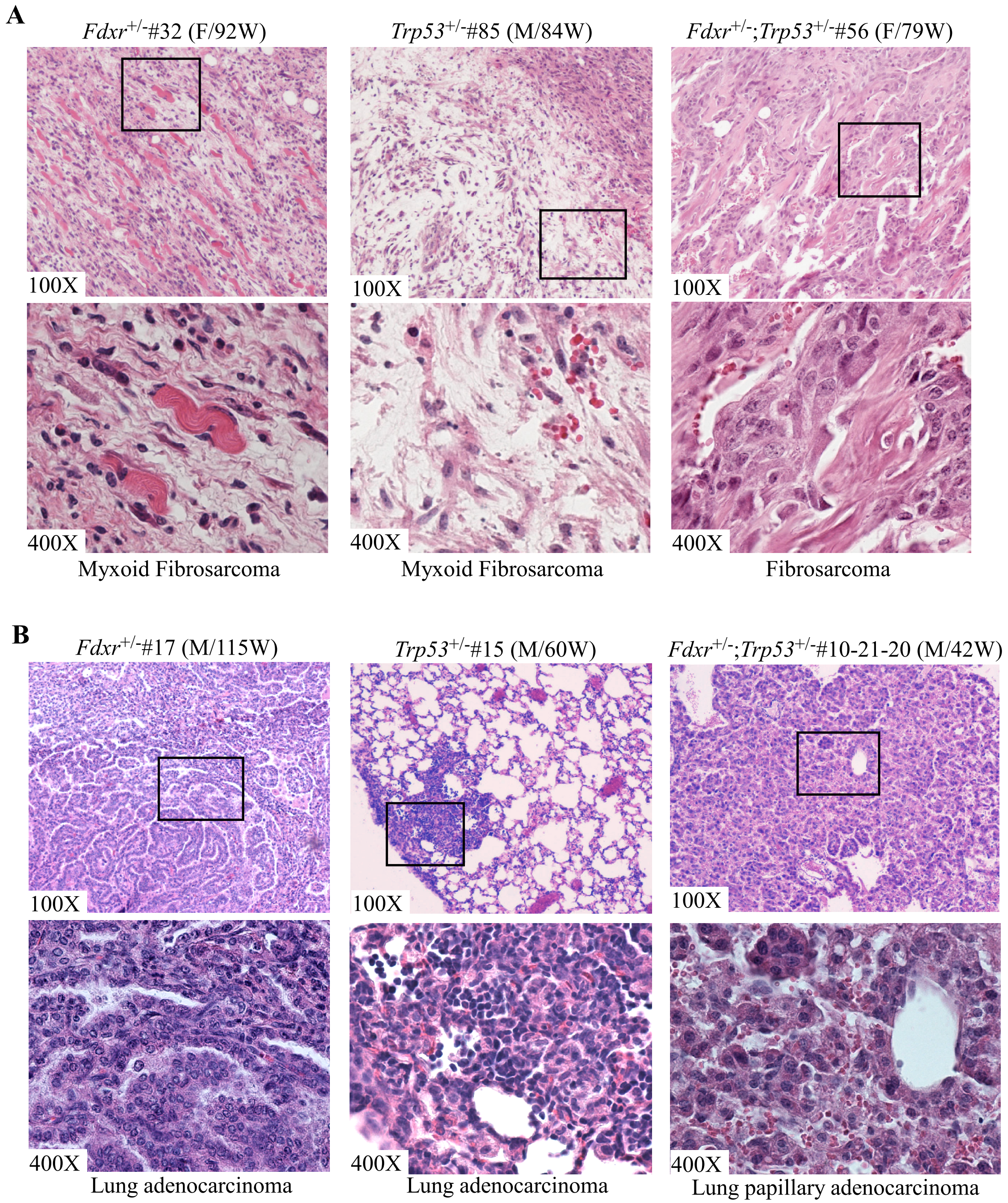

Supplementary Figure S4. Mice deficient in *Fdxr*, *Trp53*, or both are prone to spontaneous tumors. Representative images of H&E-stained fibrosarcoma (**A**) and lung adenocarcinoma (**B**) in *Fdxr*$^{+/-}$, *Trp53*$^{+/-}$ or *Fdxr*$^{+/-}$;*Trp53*$^{+/-}$ mice.
