# Supplementary material for: Ferredoxin reductase and p53 are necessary for lipid homeostasis and tumor suppression through the ABCA1–SREBP pathway
Source: Oncogene. 2022 Feb 4;41(12):1718–26. doi: 10.1038/s41388-021-02100-0 (PMC8933276; doi:10.1038/s41388-021-02100-0)
Supplement: Supplementary file 2 — Supplementary Tables S1-S8 [file 41388_2021_2100_MOESM2_ESM.pdf]

Supplementary Table S1: Wild-type (WT) mice - survival time, tumor spectrum and other abnormalities (n=32)

ID	Gender	Survival (Wks)	Tumor	Other abnormalities
5	F	134	No	No
7	F	117	No	No
16	F	100	No	No
22	F	109	No	No
25	F	109	No	No
44	F	90	No	No
55	F	104	T-LBL in Liver, thymus, salivary gland, spleen and kidney	No
64	F	120	No	No
2	M	127	No	No
3	M	117	No	Chronic hepatitis
11	M	111	N/A	Found dead
12	M	127	No	No
13	M	127	No	Normal
20	M	122	No	Normal
23	M	122	No	Normal
26	M	127	No	Chronic hepatitis and sialadenitis
34	M	124	DLBCL in liver, spleen, kidney and pancreas	No
37	M	134	No	No
62	M	128	No	No
42	M	111	N/A	Found dead
43	M	107	N/A	Found dead
45	M	133	No	No
46	M	117	N/A	Found dead
49	M	117	No	No
50	M	113	T-LBL in Thymus; DLBCL in spleen and intestine	No
52	M	101	N/A	Found dead
56	M	117	DLBCL in spleen, kidney and pancreas	No
59	M	119	DLBCL in liver, spleen	Enlarged lymph mesentery nodes
65	M	106	No	No
69	M	102	DLBCL in lung, spleen and kidney	Enlarged spleen
70	M	103	No	Enlarged thymus
71	M	90	No	No

T-LBL: T-cell lymphoblastic lymphoma; DLBCL: Diffuse large B-cell lymphoma; N/A: not applicable

*WT mice were from published studies (Zhang et al, 2017, Genes & Dev, 31:1243-56; Zhang, et al, 2019, Oncogene, 38:6256-69)

Supplementary Table S2: *Fdxr*$^{+/-}$ mice - survival time, tumor spectrum and other abnormalities (n=31)

ID	Gender	Survival (Wks)	Tumor	Other abnormalities
1	M	123	DLBCL in thymus, salivary, lung, liver, pancreas, and spleen	Liver steatosis and hepatitis
6	M	119	Not applicable	Found dead
14	M	100	Hepatocellular carcinoma; Spleen angiosarcoma	Liver steatosis
17	M	115	Lung adenocarcinoma	Liver steatosis; Thymus hyperplasia
18	M	98	Not applicable	Found dead
24	M	116	Lung carcinoma	Liver necrosis and hepatitis
27	M	115	T-LBL in thymus	Liver hepatitis; Lung chronic inflammation
21	M	114	Liver hemangioma; DLBCL in adrenal gland and spleen	EMH in spleen
33	M	112	T-LBL in thymus, kidney and spleen	No
31	M	102	T-LBL in thymus, salivary, kidney, pancreas and intestine	No
36	M	125	Liver angiosarcoma	Mildly enlarged spleen
38	M	104	No	Liver steatosis; large cell dysplasia in liver and spleen
57	M	106	No	Liver steatosis; Kidney inflammation
60	M	115	Liver hemangioma; Lung adenocarcinoma	Liver steatosis and chronic hepatitis
15	F	102	T-LBL in Thymus; Spleen angiosarcoma	Large cell hyperplasia in liver
32	F	92	Fibrosarcoma and lymphoma on left shoulder; lymphoma in salivary gland, spleen and lung	Liver chronic hepatitis
63	F	86	DLBCL in liver, thymus, spleen and pancreas	Liver steatosis and hepatitis; Lymphocytic sialadenitis; Endometrial polyp
73	F	68	DLBCL in spleen and in kidney	Lymphocytic sialadenitis; Spleen hyperplasia
40	F	100	DLBCL in spleen	Liver steatosis
41	F	39	Myeloid sarcoma in spleen; DLBCL in liver, spleen, kidney and intestine; Ovarian adenoma	Liver chronic hepatitis
47	F	60	No	Liver steatosis
48	F	100	High grade sarcoma in liver, ovary, uterus, and abdomen; DLBCL in spleen	Hepatitis
35	F	77	T-LBL in thymus	Liver steatosis Large cell hyperplasia
51	F	113	Thymus adenoma; DLBCL in salivary gland	Liver steatosis and hepatitis
53	F	90	DLBCL in liver, thymus, lung, pancreas and ovary	Liver steatosis
54	F	102	Lung adenocarcinoma	Liver steatosis
58	F	97	Hepatocellular carcinoma; Spleen myeloid sarcoma, lymphoma and hemangioma	Liver steatosis; Intestine appendiceal epiploica
29	F	108	Salivary gland and kidney plasmacytoma	Thymus hyperplasia
61	F	100	DLBCL in kidney	Thymus, spleen hyperplasia
4	F	114	DLBCL in thymus, kidney, ovary, pancreas, shoulder, and abdominal lymph nodes.	Liver steatosis with inflammation; pancreas islet cell hyperplasia
19	F	116	DLBCL in liver, pancreas, spleen, kidney, and ovary	Hepatitis and Pneumonia

T-LBL: T-cell lymphoblastic lymphoma; DLBCL: Diffuse large B-cell lymphoma; EMH: extramedullary hematopoiesis

FDXR$^{+/-}$ mice were from published studies (Zhang et al, 2017, Genes & Dev, 31:1243-56; Zhang et al, 2019, Oncogene, 38:6256-69)

Supplementary Table S3: *p53*$^{+/-}$ mice - survival time, tumor spectrum and other abnormalities (n=24)

ID	Gender	Survival (Wks)	Tumor	Other abnormalities
15	M	60	Adenocarcinoma and liposarcoma	Liver inflammation
2-1-7	M	97	Lymphoma	Hepatitis; spleen red pulp hyperplasia
85	M	84	Fibrosarcoma	Liver inflammation
2-1-4	F	62	Thymic T-cell lymphoma	No
1-4-4	M	96	DLBCL and hepatocellular carcinoma	Mild liver steatosis
1-9-15	M	31	Thymic T-cell lymphoma	No
2-16	M	91	Osteosarcoma	Liver steatosis
63	F	62	Osteosarcoma and liposarcoma	Liver steatosis
17	F	61	Fibrosarcoma	Mild liver steatosis
61-2	F	68	Lymphoma	Mild liver steatosis
24	M	36	Fibrosarcoma	Liver inflammation
72	F	57	Squamous cell carcinoma	Liver inflammation
41	M	33	Lymphoma	Liver steatosis
1-4-3	M	34	Squamous cell carcinoma	Liver inflammation and mild liver steatosis
2-3	M	64	Fibrosarcoma	Liver inflammation
70	F	68	Osteosarcoma	Liver steatosis
91	M	74	Lymphoma	Spleen pulp hyperplasia; mild liver steatosis
68	M	67	Squamous cell carcinoma	No
84	M	77	DLBCL	DLBCL in liver and spleen; liver steatosis
2-1-6	M	92	DLBCL	DLBCL in liver, lung and spleen
53	F	109	Osteosarcoma	No
18	F	48	Fibrosarcoma	Mild liver steatosis
80	M	65	Liposarcoma	No
19	F	61	No	Liver steatosis

DLBCL: Diffuse large B-cell lymphoma; N/A: not applicable; Found dead mice were excluded from tumor study

p53$^{+/-}$ mice were from a previously published study (Zhang et al, PNAS, 2014 Dec 30;111(52):18637-42)

Supplementary Table S4: *FDXR*$^{+/-}$;*p53*$^{+/-}$ mice - survival time, tumor spectrum and other abnormalities (n=29)

ID	Gender	Survival (Wks)	Tumor	Other abnormalities
9-13-8	F	40	DLBCL	-
7-17-21	F	48	DLBCL	Thymic hyperplasia; liver and kidney chronic inflammation
18	M	25	T-LBL	Liver steatosis
10-21-20	M	42	Lung papillary adenocarcinoma; DLBCL; benign teratoma	Atrophic thymus; liver and kidney chronic inflammation; spleen EMH; inflamed dermoid cysts on reproductive organ; chronic sialadenitis
8-13-20	M	94	High grade pleomorphic fibrosarcoma	Mild thymic hyperplasia, liver chronic inflammation; spleen hyperplasia/EMH; chronic sialadenitis
3-24-25	M	86	Lymphoma	Mild thymic hyperplasia; spleen hyperplasia/EMH; lung, liver and kidney chronic inflammation; steatosis; chronic sialadenitis
21	F	73	Fibrosarcoma; Splenic Follicular lymphoma	Thymic hyperplasia; liver chronic inflammation; steatosis; chronic sialadenitis
33	M	57	Lymphoma	Mild thymic hyperplasia; spleen hyperplasia/EMH; liver, pancreas and kidney chronic inflammation; atypical follicular hyperplasia in LN; steotosis; chronic sialadenitis
67	F	60	DLBCL	Atrophic thymus; liver and kidney chronic inflammation; spleen white pulp atypical follicular hyperplasia/red pulp EMH; chronic sialadenitis
71	F	62	DLBCL	Spleen EMH; mild steatosis
83	F	56	DLBCL	Thymic hyperplasia; chronic sialadenitis; liver necrosis; steatosis
85	F	61	DLBCL	Thymic hyperplasia; spleen hyperplasia/EMH; liver and kidney chronic inflammation; chronic sialadenitis; mild steatosis
56	F	79	Fibrosarcoma	Thymic hyperplasia; spleen hyperplasia/EMH; chronic sialadenitis
35	M	36	Benign teratoma	Inflamed dermoid cysts on reproductive organ; thymic and splenic hyperplasia; spleen EMH; chronic sialadenitis
9-14-33	F	47	Adenocarcinoma	Thymic hyperplasia; sialadenitis and reactive follicular hyperplasia; marked periportal inflammation with hepatitis in liver; spleen hyperplasia/EMH, increased gut associated lymphoid follicles; steatosis
42	M	52	No	Thymic hyperplasia; sialadenitis; marked periportal inflammation with hepatitis in liver; spleen hyperplasia/EMH; increased gut associated lymphoid follicles; reactive follicular hyperplasia in LN
4	F	56	Lymphoma; Pleomorphic sarcoma with angiosarcoma; benign hemangioma	Splenic EMH; mild steatosis
24	F	62	Pleomorphic sarcoma	Thymic hyperplasia; lung and liver focal perivascular chronic inflammation; spleen EMH
5-7-42	F	71	Pleomorphic sarcoma with angiosarcoma	Thymic hyperplasia; liver focal perivascular chronic inflammation; spleen follicular hyperplasia/EMH; pulmonary venous vasculopathy with peribronchitis; sialadenitis; reactive lymphoid hyperplasia; mild steatosis
90	M	71	Hepatocellular carcinoma; Histiocytic sarcoma	Liver necrosis; pulmonary venous vasculopathy with peribronchitis
27	M	84	Squamous cell carcinoma; Osteosarcoma	Spleen follicular hyperplasia/EMH; steatosis
69	F	50	Squamous cell carcinoma	Thymic hyperplasia; liver and kidney chronic inflammation; spleen follicular hyperplasia/EMH; sialadenitis
52	F	52	DLBCL	Thymic hyperplasia; liver and kidney focal chronic inflammation; spleen follicular hyperplasia/EMH; sialadenitis
74	M	74	Fibrosarcoma; Basaloid squamous cell carcinoma	Spleen follicular hyperplasia/EMH
63	F	72	DLBCL	Liver steatohepatitis
22	F	72	N/A	Found dead
15	F	70	N/A	Found dead
26	F	73	N/A	Found dead
59	M	81	N/A	Found dead

T-LBL: T-cell lymphoblastic lymphoma; DLBCL: Diffuse large B-cell lymphoma; EMH: extramedullary hematopoiesis

Supplementary Table S5. Complete Blood Count (CBC) in WT, *Fdxr*$^{+/-}$, *Trp53*$^{+/-}$ and *Fdxr*$^{+/-}$;*Trp53*$^{+/-}$ mice

A. The levels of RBC, RDW, Hemoglobin and Hematocrit in WT, *Fdxr*$^{+/-}$, *Trp53*$^{+/-}$ or *Fdxr*$^{+/-}$;*Trp53*$^{+/-}$ mice

	RBC (M/μL)	RDW (%)	Hemoglobin (g/dL)	Hematocrit (%)
WT (n=5)	10.14±0.22	21.00±2.50	12.95±0.65	42.55±0.65
Fdxr$^{+/-}$ (n=5)	10.05±0.16	18.04±0.13	13.40±0.26	42.50±0.96
Trp53$^{+/-}$ (n=5)	10.09±0.37	19.05±1.18	13.48±0.43	42.85±1.61
Fdxr$^{+/-}$;*Trp53*$^{+/-}$ (n=5)	9.61±0.16	18.19±0.24	12.99±0.25	41.51±0.80

RBC: Red Blood Cell; RDW: Red cell Distribution Width

B. The levels of MCV, MCH, MCHC, Platelets and MPV in WT, *Fdxr*$^{+/-}$, *Trp53*$^{+/-}$ or *Fdxr*$^{+/-}$;*Trp53*$^{+/-}$ mice

	MCV (fL)	MCH (pg)	MCHC (g/dL)	Platelets (K/μL)	MPV (fL)
WT (n=5)	41.95±0.25	12.75±0.35	30.45±1.05	1894.00±33.00	5.45±0.05
Fdxr$^{+/-}$ (n=5)	42.30±0.75	13.32±0.20	31.52±0.21	1479.40±79.82	5.40±0.11
Trp53$^{+/-}$ (n=5)	42.20±0.54	13.35±0.26	31.48±0.32	1560.75±443.97	5.45±0.16
Fdxr$^{+/-}$;*Trp53*$^{+/-}$ (n=5)	43.22±0.44	13.52±0.15	31.30±0.40	1558.44±136.66	5.49±0.09

MCV: Mean Corpuscular Volume; MCH: Mean Corpuscular Hemoglobin;
MCHC: Mean Corpuscular Hemoglobin Concentration; MPV: Mean Platelet Volume

C. The levels of Neutrophil, Lymphocyte, Monocyte, Eosinophil and Basophil in WT, *Fdxr*$^{+/-}$, *Trp53*$^{+/-}$ and *Fdxr*$^{+/-}$;*Trp53*$^{+/-}$ mice

	WBC (K/μL)	WBC (100%)				
		Neutrophil (%)	Lymphocyte (%)	Monocyte (%)	Eosinophil (%)	Basophil (%)
WT (n=5)	5.77 ± 1.63	58.04± 17.82	37.30 ±13.10	2.86 ±0.15	1.18 ±0.31	0.64±0.37
Fdxr$^{+/-}$ (n=5)	3.72 ± 0.78	34.83± 6.97	57.69 ±6.34	5.68 ±1.02	1.52 ±0.75	0.55±0.32
Trp53$^{+/-}$ (n=5)	4.97 ± 0.99	37.43 ±10.73	56.57 ±10.22	3.91 ±0.77	1.54 ±0.42	0.56±0.49
Fdxr$^{+/-}$;*Trp53*$^{+/-}$ (n=5)	4.54 ± 1.51	41.74 ±5.22	54.92 ±4.26	4.88 ±0.46	1.42 ±0.46	0.36±0.29

Supplementary Table S6. The oligos used for generation of sgRNA expression vectors

Name	Sequence
Human p53 sgRNA#1	Sense: 5'-caccgACTGGGACGGAACAGCTTTG3'<br>Antisense: 5'-aaacCAAAGCTGTTCCGTCCCAGTc-3'
Human p53 sgRNA#2	Sense: 5'-caccgTCTGTGCGCCGGTCTCTCCC-3'<br>Antisense: 5'-aaacGGGAGAGACCGGCGCACAGAc-3'
Human FDXR sgRNA#1	Sense: 5'- caccgCCTTCGTGGGCTGGTACAAC-3'<br>Antisense: 5'-aaacGTTGTACCAGCCCACGAAGGc-3'
Human FDXR sgRNA#2	Sense: 5'-caccgCCGCCTAGCAGTCACTAGAC-3'<br>Antisense: 5'-aaacGTCTAGTGACTGCTAGGCGGc-3'

Supplementary Table S7. The primers used for mouse and cell lines genotyping

Name	Sequence
Fdxr-KO mouse genotyping	Sense: 5'-GGCATCTGGAGGTACTAGGAAG-3'<br>Antisense: 5'-AGTTGCTGGCTTGGTCTGTCTG-3'<br>Antisense: 5'-CCCCCACAGAACTCTATTGCTC-3'
Trp53-KO mouse genotyping	Sense: 5'-ACGCCAGACTTTGCTGAGTT-3'<br>Antisense: 5'-GAGGAGCCAGGAGAGGACTT-3'<br>Antisense: 5'-CGATGATGTGGCTTTGAAGA-3'
Human *FDXR*-KO cell line genotyping	Sense: 5'-AGGGACAGCCTGGAGACATT-3'<br>Antisense: 5'-GGGGTGTCTTTGGGAAACAT-3'

Supplementary Table S8. siRNA Oligonucleotides

Name		Sequence
Scrambled siRNA		5'-GCAGUGUCUCCACGUACUAdTd-3'
siFDXR-human	#1:	5'-CACCAUUAAGGAGCUUCGGdTdT-3'
	#2:	5'-GCUCAGCAGCAUUGGGUAUdTdT-3'
sip53-human	#1:	5'-GAAAUUUGCGUGUGGAGUAdTdT-3'
	#2:	5'-GCACAGAGGAAGAGAAUCUdTdT-3'
